# Surgical Cases

**Published:** 1853-11

**Authors:** R. D. Mussey

**Affiliations:** Professor of Operative Surgery in the Miami Medical College, at Cincinnati, Ohio


From the American Journal.

*Surgical Cases. Aneurismal Tumours upon the Ear, successfully treated by the Ligation of both Carotids.—Recto Vaginal Fistula, cured by Operation.* By R. D. Mussey, M.D., Professor of Operative Surgery in the Miami Medical College, at Cincinnati, Ohio.

Case I. *Aneurismal Tumours upon the ear treated by Ligation of both Carotids.*—Early in November last, Luther Gordon,

æ. 19, accompanied by his physician, Dr. Kramer, came from Indiana, with his head bound up, to this city, on account of aneurismal tumours upon his left ear, and was admitted into St. John's Hospital.

The cavity of the concha was occupied by a pouch which rose above the level of the antitragus, and another covering the tragus and extending some way anterior to it, and pushing outward, was as large as a middling sized nutmeg. Continuous with the upper part of this was a considerable elevation of the integument which covered the scaphoid fossa, and an inch and a half of the fossa innominata. Below the root of the ear, in the depression between the mastoid process and the ramus of the jaw, and partially covered by the lobulus, was a globular tumour of the same character, as large as a moderate-sized Isabella grape. All these tumours or pouches, were elastic, and compressible almost to obliteration, pulsated strongly, and seemed to have a communication with each other, like the portions of an arterial varix. The whole circumference of the ear was larger than that of the other, and its integuments everywhere hypertrophied.

L. G. was of medium stature, with auburn hair and hazel eyes, and, although somewhat delicate in appearance, had enjoyed, from childhood, a pretty uniform health. From birth there was a cutaneous nævus in front of the left ear, but it attracted no particular attention. About eight years ago small elevations of the integument were observed at the points already described as the site of the tumours, in which pulsation was perceptible, especially after exercise. This, together with the size of the tumours, slowly increased, until, a month before he came here, the posterior extremity of the pouch occupying the fossa innominata burst open, causing alarming hemorrhage. This was suppressed by compression; and, subsequently, when the bandage and compresses were removed, the crust covering the opening gave way, and a pulsating jet of arterial blood followed.

With reference to the treatment of this case, the most promising course which presented itself, was the ligation of one or both carotids. The success which followed the tying of the primitive carotid, by Mr. Travers, in 1809,* for "aneurism by anastomosis of the orbit;" and in a similar case by Mr. Dalrymple, in 1813; † and also the tying of both carotids, by Dr. J. Mason Warren, in a remarkable case of vascular tumour of the mouth, face, and neck, in 1846,‡ afforded encouragement for this procedure; yet the case I had in 1829 § in which I tied both carotids for a large vascular

* Medico-Chirurg. Trans. vol. ii.

† Ibid. vol. vi.

‡ Amer. Jour. Med. Science, vol. ii. New Series, p. 281, 1846.

§ Ibid. vol. v. p. 316, 2829.

pulsating tumour on the vortex of the head, not having been cured until the tumour was dissected away, left room for doubt whether, in the present instance, the ligature of both carotids even might not fail of accomplishing the end desired. I determined, however, to resort to the application of a ligature to one of these vessels, possibly both. The patient had been kept chiefly on farinaceous food since the first outbreak of the hemorrhage, and it was now enjoined upon him to live wholly without animal food until the operation.

On the 18th of November, I tied the left carotid. The pulsation in the tumours ceased on tightning the ligature, and did not afterwards return. His food was strictly farinaceous, with water for his only drink. After the lapse of ten days, a little milk was allowed. No unpleasant symptom occurred, except that when he began to sit up, which he was permitted to do in twelve days, he complained of indistinctness of vision in the left eye. It continued for several days, though less and less marked, till it ultimately subsided altogether. This symptom, indicating a defective supply of blood to the visual apparatus, has been sometimes observed, but I had not myself before noticed it in either of the six cases in which I had applied a ligature to the common carotid. A slow reduction of the tumours took place; but, as it was quite doubtful whether a cure would follow, I proceeded, in four weeks, to ligate the right carotid. A slight effect was observed on the vision of the right eye when the patient began to sit up, similar to what had taken place with the other.

The two operations were performed while the patient was asleep from the inhalation of a mixture of chloroform, one part by measure, and washed sulphuric ether, two parts. Both arteries were tied just below the crossing of the omohyoid muscle. One ligature came away in sixteen days, the other in twenty. After the second operation the reduction in size of the tumours was much more rapid. In about three weeks, collodion was applied and repeated every two or three days. This seemed very much to promote the contraction of the pouches, and on the 28th of January, viz., *seven* weeks from the last operation, L. G. left for home with scarcely a vestige of the tumours remaining. I considered the result of the operations to be a permanent cure.

The last of April, three months after the patient went home, one of his physicians, residing near him, called on me, and gave the assurance that there were no remains of the swelling, and that he regarded the case as perfectly cured.

Case II. *Recto-Vaginal Fistula.*—Mrs. G., æ. 28, of fair complexion and delicate appearance, but possessing a pretty good constitution, apparently free from hereditary tendency to disease, was married between five and six years since. Being subject to costiveness, the recto-vaginal wall, under the influence of undue pressure, gave way some time after marriage and a fistulous opening remained. This was somewhat enlarged during labor with her only child, which was born some two years after matrimony. Being very cleanly in her habits, Mrs G. was able to keep herself comfortable when the feces were of a firm consistence, but when diarrhœa, or a state approaching it, existed, a considerable portion of the contents of the rectum passed through the vagina. All along, the monthly evacuation was uninterrupted, and the state of the bowels was regulated by aperients and injections.

On the 25th of March, 1853, I performed the first operation, which consisted in a division of the sphincter ani on one side, the object of which was to promote the contraction of the fistula by allowing the feces to pass through the anus without effort. Before this wound was quite healed, I proceeded, on the 20th of April, assisted by my son, Dr. Wm. H. Mussey, Dr. A. M. Slocum, and Dr. Logan, to the second operation. The hair having been removed from around the anus and posterior part of the vulva, the patient was put into the anæsthetic state by the mixture of chloroform and ether, and placed in the position usually chosen for lithotomy, the lower limbs being supported by assistants. A bivalve speculum was passed into the anus, while the sides of the vulva were drawn aside. In this state of tension of the parts, the fistula, brought fully to view, was sufficiently large to admit the two fingers. It was slightly oval shaped, its longest diameter forming an angle with the median line. The edges of the opening were freshened by a straight, narrow, sharp-pointed bistoury, and brought in contact and sustained by the *clamp suture* of Dr. Sims, of Alabama. A piece of elastic gum catheter was secured in the urethra; and the urine, the whole of which passed through the instrument, was received by a sponge, or a folded cloth. The catheter was removed and cleaned every second or third day, and returned to its place, or replaced by a new one. The patient lay chiefly on her back, sometimes upon her side. She slept well the night after the operation, after taking the eighth of a grain of the sulphate of morphia. She took this dose but once more during her confinement; in a few instances, when a little restless at evening, she took a teaspoonful of the fluid extract of valerian. She generally slept well at night. The pulse was scarcely, if at all, accelerated; there was no thirst; the tongue was clean; and there was no headache, except a little in the mornings after morphia or valerian had been taken.

This undisturbed state of the system was to be attributed, in a great measure, to the unstimulating and spare diet which was persevered in. Up to the eighteenth day from the second operation, she lived on *two* to *two and a half crackers a day.* The whole weight of this solid food was *less than five ounces* ; the only drink was cold water. On the eighteenth day, a gill of milk for the twenty-four hours was allowed in addition. The vagina was daily injected with water, and mopped dry. On the *seventh* day the stitches were cut out, and the wound was found united through the whole extent. The catheter was left out on the *eighteenth* day, and the patient allowed to be bolstered up a little in bed for half an hour, which was repeated two or three times a day afterwards.

On the *twenty-fourth day* a motion of the bowels was procured by two drachms of castor-oil made into an emulsion with mucilage, and given every three hours until it operated. Nothing had passed the bowels all this while, except occasionally a small quantity of gas. From this time Mrs. G. took more food; was soon able to sit up all day ; and left for home on the *twenty-second* of May, four and a half weeks after the operation. Two weeks after she had returned home, she wrote that she was perfectly well in all respects.

Dr. Sims* is well entitled to the thanks of the profession for having introduced what he calls the *clamp suture*, in the treatment of vesico-vaginal fistula, consisting of two cylinders of silver, or lead, perforated at several points, for the passage of pieces of small silver wire, which are to supply the place of thread, and which are to be prevented from slipping by perforated shot carried down upon them, pressed against the cylinders, and kept in place by being firmly pinched with pliers. Dr. S. makes his cylinders one line in diameter, and his wires of the size of a horse-hair.

In the case of Mrs. G., I used leaden cylinders, a line and a half in diameter, believing that they would be less liable to become imbedded, and to cause ulceration in the soft parts against which they are pressed ; they were perforated too at distances of *one-fifth* of an inch, instead of *one-third*, or more, of an inch, as practised by Dr. S. I see no objection to the stitches being within the fifth of an inch of each other, inasmuch as there is little, if any, tendency to suppuration around the wires ; and there seems to me to be this advantage from the near stitches, viz., that the parts intermediate to them may be brought into sufficiently firm contact for adhesion, with a less amount of pressure, and of course with less liability to strangulation of the vessels of the parts included in the suture. Dr. Thomas, in the eastern part of Ohio, now of Pittsburg, Pa., who treated a case of vesico-vaginal fistula with entire success, placed his stitches in the clamp suture about the fifth of an inch apart. The wire which I employed in the recto-vaginal fistula, was not far from twice the diameter of a horse hair. I suppose that the shot compressed upon it is a little less liable to

---

* American Journal of Medical Science, Jan., 1852.

slip than upon one only half the diameter. The stitches were entered about one-third of an inch from the cut edge of the opening and carried as deep as possible, without passing through the mucous lining of the rectum. When the suture was removed on the seventh day, it was found that a slight ulceration existed where the extremity of one of the cylinders lay. This was healed in a few days.

Cincinnati, June 20, 1853.

P. S. I saw Mrs. G. on the 30th of July, more than three months after the operation, in a state of perfect soundness of health.

August 25, 1853.

